# Lower Adherence to a Mediterranean Diet Is Associated with High Adiposity in Community-Dwelling Older Adults: Results from the Longevity Check-Up (Lookup) 7+ Project

**DOI:** 10.3390/nu15234892

**Published:** 2023-11-23

**Authors:** Stefano Cacciatore, Giordana Gava, Riccardo Calvani, Emanuele Marzetti, Hélio José Coelho-Júnior, Anna Picca, Ilaria Esposito, Francesca Ciciarello, Sara Salini, Andrea Russo, Matteo Tosato, Francesco Landi

**Affiliations:** 1 Department of Geriatrics, Orthopedics and Rheumatology, Università Cattolica del Sacro Cuore, L.go F. Vito 1, 00168 Rome, Italy; stefano.cacciatore01@icatt.it (S.C.); giordana.gava01@icatt.it (G.G.); emanuele.marzetti@policlinicogemelli.it (E.M.); coelhojunior@hotmail.com.br (H.J.C.-J.); ilaria.esposito1@unicatt.it (I.E.); francesco.landi@unicatt.it (F.L.); 2 Fondazione Policlinico Universitario “Agostino Gemelli” IRCCS, L.go A. Gemelli 8, 00168 Rome, Italy; picca@lum.it (A.P.); francesca.ciciarello@policlinicogemelli.it (F.C.); sara.salini@policlinicogemelli.it (S.S.); andrea.russo1@policlinicogemelli.it (A.R.); matteo.tosato@policlinicogemelli.it (M.T.); 3 Department of Medicine and Surgery, LUM University, SS100 km 18, 70100 Casamassima, Italy

**Keywords:** aging, body composition, body fat, relative fat mass, nutrition, obesity, Medi-Lite, healthy diet, lifestyle, behavioral interventions

## Abstract

High adiposity impacts health and quality of life in old age, owing to its association with multimorbidity, decreased physical performance, and frailty. Whether a high adherence to a Mediterranean diet (Medi-Diet) is associated with reduced body adiposity in older adults is unclear. The present study was conducted to assess the prevalence of high adiposity in a large sample of community-dwelling older adults. We also explored the relationship between whole-body adiposity estimated through relative fat mass (RFM) and Medi-Diet adherence. Data were obtained from the Longevity Check-up 7+ (Lookup7+) project database. RFM was estimated from anthropometric and personal parameters using a validated equation. RFM was categorized as high if ≥40% in women and ≥30% in men. Information on diet was collected using a food frequency questionnaire, while Medi-Diet adherence was assessed through a modified version of the Medi-Lite scoring system. Analyses were conducted in 2092 participants (mean age 73.1 ± 5.9 years; 53.4% women). Mean RFM was 39.6 ± 5.14% in women and 29.0 ± 3.6% in men. High adiposity was found in 971 (46.4%) participants and was more frequent in those with a low (54.2%) or moderate (46.4%) Medi-Diet adherence compared with the high-adherence group (39.7%, *p* < 0.001). Logistic regression indicated that older adults with high Medi-Diet adherence were less likely to have a high RFM. Other factors associated with a greater risk of having high adiposity were older age, female sex, and physical inactivity. Our findings support an association between healthy lifestyles, including a greater adherence to a Mediterranean-style diet, and lower body adiposity in older adults.

## 1. Introduction

Excessive fat accumulation is associated with negative health outcomes and is a public health concern at all life stages [1,2]. The World Health Organization estimates that the global prevalence of obesity has increased threefold since 1975, affecting more than 650 million individuals (13%) worldwide in 2016 [2]. According to the World Obesity Federation, more than half of the global population will be overweight or obese in the next 12 years [3]. Adipose tissue accumulation, infiltration, and dysfunction are responsible for endocrine, cardiometabolic, and functional alterations [4,5,6]. Body mass index (BMI) is the most used metric to assess adiposity in the general population. BMI values greater than 25 kg/m^2^ and 30 kg/m^2^ (which indicate overweight and obesity, respectively) are associated with ncreased morbidity and mortality [7,8]. However, recent evidence suggests that BMI may not possess sufficient sensitivity for identifying excess fat at the individual level [9,10]. Furthermore, the association between BMI-defined obesity and negative health outcomes in older adults is controversial. According to a recent systematic review, greater BMI values were negatively associated with short- and long-term all-cause mortality in older adults, especially in those with chronic diseases or facing an acute medical event. This counterintuitive finding could potentially be attributed to the lack of body composition information provided by BMI, as well as the weaker correlation between BMI and percentage of body mass or fat mass index [11]. Indeed, a meta-analysis of 32 studies comprising 31,968 individuals from 12 countries found that commonly used BMI cutoffs for diagnosing obesity failed to identify almost half of those with high adiposity as assessed by body composition reference techniques (e.g., dual energy X-ray absorptiometry (DXA), air-displacement plethysmography, hydrostatic weighing) [9].

The availability of reliable and easy-to-apply measures of body fat is particularly relevant to older adults, as aging is often accompanied by fat accumulation at the expenses of lean body mass [12]. Novel whole-body fat estimators based on anthropometric measures have been proposed and validated [13,14,15]. Among them, relative fat mass (RFM) is a simple equation based on the ratio of height to waist circumference that was developed by Woolcott and Bergman [15] using data from National Health and Nutrition Examination Survey (NHANES) 1999–2004 and 2005–2006. RFM proved to be more accurate than BMI for estimating whole-body adiposity and reducing obesity misclassification in adults of different ethnicities, especially among older individuals [15,16,17]. In the general population, a high RFM has been associated with incident cardiovascular disease and diabetes as well as mortality [18,19,20,21]. An RFM greater than 26% in men and equal to or greater than 38% in women was related with incident atrial fibrillation, heart failure, and coronary artery disease in middle-aged individuals from the Dutch Lifelines Cohort Study over a four-year follow-up. [18]. In the PREVEND (Prevention of Renal and Vascular End-Stage Disease) study, several indices of adiposity were evaluated as potential predictors of incident heart failure during 11 years of follow-up in community-dwelling adults [21]. When compared with other adiposity indices (i.e., body circumference index, weight-adjusted waist index, and body shape index), RFM had the strongest association with heart failure with either preserved or reduced ejection fraction [21]. In the PREVEND cohort, RFM was also associated with the incidence of type 2 diabetes [19], which was validated in two independent Dutch cohorts (i.e., Lifelines and Rotterdam cohorts) [19]. In adults with type 2 diabetes, RFM was negatively associated with trabecular bone score and exhibited the highest predictive value for the likelihood of damaged bone microarchitecture compared with insulin resistance indices and hormonal indicators [22]. Recent evidence from the Moli-sani study showed that participants in the highest RFM quartile had an increased mortality risk, which was mediated by forced expiratory volume in 1 s (FEV1) and blood glucose and cystatin C levels [20]. In older adults with obesity, RFM has shown to be sensitive to changes following a 12-month weight loss intervention based on exercise and energy restriction [23]. A remarkable agreement was observed between RFM and direct measures of adiposity by DXA or magnetic resonance imaging (MRI), particularly among old women [23].

Physical inactivity and unhealthy dietary habits are the main modifiable contributors to excess adiposity [24]. The Mediterranean diet (Medi-Diet) is considered a healthy dietary model [25,26], owing to its well-known beneficial effects on several chronic conditions, including cardiovascular diseases [27], type 2 diabetes mellitus [28], cognitive decline [29,30], sarcopenia [31], osteoporosis [32], osteoarthritis [33,34], as well as quality of life [35]. Medi-Diet is characterized by a high intake of cereals, vegetables, fruits, and legumes, a modest intake of milk and dairy products, and sporadic consumption of meat and animal fats [36,37]. Several studies have highlighted a possible role for Medi-Diet as a suitable strategy for preventing and/or managing obesity [38,39]. However, most of the available evidence was gathered in young or middle-aged individuals, while less is known on the effects of a Mediterranean diet on body adiposity in older adults [40,41,42]. Furthermore, no studies have explored the association between adherence to a Mediterranean diet and anthropometry-based indices of body adiposity in old individuals.

The aim of this study was to assess the prevalence of high adiposity estimated by RFM in a large sample of Italian community-dwelling older individuals and explore its association with adherence to a Medi-Diet.

## 2. Materials and Methods

The data used in this study were collected within the Longevity Check-up 7+ (Lookup 7+) project. For the present investigation, analyses were performed using data on participants 65 years or older. The Lookup 7+ survey is an ongoing cross-sectional study carried out by the Department of Geriatrics, Orthopedics, and Rheumatology at the Università Cattolica del Sacro Cuore and the Fondazione Policlinico Universitario “Agostino Gemelli” IRCCS in Rome, Italy. The complete description of the study protocol can be found in other sources [43,44]. Candidate participants are considered eligible if they are 18 years or older. Self-reported pregnancy, refusal to perform capillary blood testing for the measurement of total cholesterol and glycemia, and unwillingness or inability to provide a written informed consent are exclusion criteria. Participants are recruited in public places (such as shopping malls, pharmacies, and supermarkets) and events, as well as during health prevention campaigns. Cities of varying sizes are selected to obtain a complete geographic characterization of Italian mainland and major islands. Cities are categorized as small, with a population of less than 100,000 people, medium-sized, with a population ranging from 100,000 to 250,000 residents, and large, with a population exceeding 250,000 inhabitants. The selection of locations is determined according to the availability of potential volunteers who can be approached. To provide adequate representation of the sociodemographic characteristics of residents, in large cities (e.g., Rome, Milan, Naples, Genoa, Bologna, Catania), activities are carried out in different places. Before enrolling, every participant is required to provide a signed informed consent. The study protocol was authorized by the Ethics Committee of the Università Cattolica del Sacro Cuore (protocol no.: A.1220/CE/2011).

At each location, assessments take place within a quiet and socially distanced moveable medical booth. To ensure consistency, all assessments are conducted in a standardized sequence: lifestyle interview, measurement of blood pressure, measurement of body weight and height, capillary blood testing for total cholesterol and glycemia, physical performance tests. As previously reported [43], the average completion time for each visit is 12.8 ± 1.6 min, with no significant differences among age groups, sexes, or settings.

### 2.1. Anthropometry and Lifestyle Habits

A questionnaire on dietary and other lifestyle habits was administered by trained personnel to collect information on personal characteristics and modifiable risk factors for chronic diseases [44]. Body weight and height were measured using an analog column scale with integrated stadiometer. The BMI was computed as the square of height (in m^2^) divided by body weight (in kg). An anthropometric tape was used to measure the participant’s waist circumference (in cm) as they stood with their feet together, head straight, eyes forward, and their arms at their sides. The measurement was taken at the midpoint between the last floating rib and the highest point of the iliac crest. Two categories (i.e., active or past/never smoker) were used to define smoking status. Regular engagement in physical activity or exercise was operationalized as consistent participation in either structured or non-structured activities, with a frequency of at least two sessions per week, each lasting for a minimum of 30 min, over the course of the preceding year. Individuals who did not engage in any form of physical activity or did not meet the frequency and/or duration specified above were classified as physically inactive [45,46].

### 2.2. Relative Fat Mass

Woolcott and Bergman’s equation [15] was used to estimate RFM, as follows:

$$\mathrm{RFM}=64-\left(20\times \frac{\mathrm{height}\;(\mathrm{cm})}{\mathrm{waist}\;\mathrm{circumference}\;(\mathrm{cm})}\right)+(12\times sex),$$

where sex = 0 for men and 1 for women.

High adiposity was operationalized as having RFM values ≥40% in women and ≥30% in men [47]. RFM cutoff values were established using cut points associated with a higher risk of all-cause mortality in the National Health and Nutrition Examination Survey (NHANES) 1999–2014 [47]. According to a receiver operating characteristic (ROC) analysis, RFM showed a high accuracy in discriminating adult individuals with DXA-defined obesity in both men and women (C-statistics = 0.91, 95% confidence interval: 0.91–0.92) [47].

### 2.3. Adherence to a Mediterranean Diet and Daily Energy Intake

Nutritional data were obtained using a simplified food frequency questionnaire (FFQ) [48]. The survey evaluated the frequency with which respondents consumed a specified portion size of 12 different foods on a weekly basis [49]. The food items explored were fish, meat and derivatives (e.g., cured meat), eggs, milk and dairy products (e.g., cheese, yogurt), pasta and bakery products (e.g., bread, pizza), rice, legumes, vegetables, and other cereals (e.g., oat, rye, spelt). The Italian standard portion reference was used to estimate portion size. Adherence to a Medi-Diet was determined through a modified version of the Medi-Lite scoring system [31,50,51]. The Medi-Lite score considers nine food categories. A score from 0 (lowest intake) to 2 (highest consumption) was assigned to food items usually included in a Medi-Diet (i.e., cereals, fruits, legumes, fish, and vegetables). For dietary categories not included in a typical Medi-Diet (e.g., meat and meat derivatives), 2 points were allocated to the lowest intake, 1 point to an intermediate consumption, and 0 points to the highest consumption. In the case of olive oil, 2 points were assigned for daily usage, 1 point for regular use, and 0 points for occasional consumption. Since alcohol use was not recorded, the corresponding item was not considered for the calculation of the Medi-Lite score. As a result, the maximum score achievable was 16 instead of 18. Details on Medi-Lite scoring have been published elsewhere [31]. A summary score of 8 points or lower indicated a low Medi-Diet adherence, a score ranging from 9 to 11 was classified as moderate adherence, and a score of 12 or higher indicated high Medi-Diet adherence.

Mean daily energy intake was calculated as the sum of calorie content of a standard portion of individual food items listed in the FFQ, divided by seven (the days of a week). Calculations were based on calorie values reported in the online database of the Italian Center for Research on Foods and Nutrition (CREA) [52].

### 2.4. Statistical Analysis

Personal and anthropometric characteristics of participants according to their Medi-Diet adherence were compiled using descriptive statistics. The normal distribution of continuous data was evaluated using the Kolmogorov–Smirnov test. Means ± standard deviations were used to summarize continuous variables, whereas categorical data were expressed as absolute frequencies and percentages of the total. Comparisons among categories of adherence to a Mediterranean diet (i.e., low, moderate, high) for continuous variables were performed by one-way analysis of variance (ANOVA), with Bonferroni’s post hoc test as needed. Chi-square statistics with Bonferroni correction for multiple comparisons were used for categorical variables. Logistic regression models were employed to examine the relationship between adherence to a Medi-Diet and high adiposity. Initially, crude odds ratios were calculated together with their corresponding 95% confidence intervals. Afterward, logistic regression models were built with adjustments for age, sex, and other factors associated with high adiposity at univariate analysis. The R software version 4.2.3 R (Core Team, Vienna, Austria) was used for all analyses.

## 3. Results

A total of 4720 participants aged 65 years or older were recruited across Italy between 1 June 2015 and 31 January 2023. Of them, 2628 were excluded due to missing values in the variables of interests (2583 for waist circumference and 1638 for nutrition data). A final sample of 2092 participants was considered for the present investigation. Personal characteristics of excluded participants were not significantly different from those with complete data. The mean age of the study population was 73.1 ± 5.9 years and 1117 (53.4%) were women. Adherence to a Medi-Diet was low in 346 (19.7%) participants, moderate in 1198 (57.3%), and high in 481 (23.0%). Table 1 shows the main characteristics of participants categorized according to their adherence to a Medi-Diet. Individuals with a low Medi-Diet adherence were predominantly women and were more frequently active smokers than in the other two groups. Daily energy intake was higher in participants with high Medi-Diet adherence than in those with moderate or low adherence. BMI values and waist circumference were lower in participants with a high Medi-Diet adherence. Mean RFM was 39.6 ± 5.1% in women and 29.0 ± 3.6% in men and was significantly lower in individuals with a high Medi-Diet adherence. A high adiposity was found in 971 (46.4%) participants and was more frequent in those with a low or moderate Medi-Diet adherence than in the high-adherence group.

In the unadjusted logistic regression model, both moderate and low Medi-Diet adherence were associated with a greater risk of high adiposity compared with high Medi-Diet adherence (Table 2).

The association remained significant after adjusting for age and sex (Model 1). After controlling for potential confounders (age, female sex, active smoking, daily energy intake, engagement in physical activity), moderate and low Medi-Diet adherence were independently associated with a greater risk of high adiposity (Model 2). Predictors of high adiposity were age and female sex in both Model 1 and Model 2 (Table 2). Engagement in physical activity was associated with a reduced risk of high adiposity in Model 2. No significant associations were found between high adiposity and daily energy intake or active smoking (Table 2).

## 4. Discussion

In the present study, we investigated the prevalence of high adiposity estimated by RFM in a large cohort of Italian older adults living in the community and explored its association with adherence to a Mediterranean-style dietary pattern. Participants with a high Medi-Diet adherence had a lower frequency of high adiposity than those with moderate or low adherence. In logistic regression models, a significant association between high adiposity and Medi-Diet adherence was found after adjusting for potential confounders. Older age and female sex were predictors of high adiposity, while engagement in physical activity was associated with lower odds of having high adiposity.

The role of Medi-Diet in the prevention of adipose tissue accumulation is well documented in the literature [38,53]. A large cross-sectional study in 3162 Spanish adults found a significantly lower risk of obesity in those with a higher Medi-Diet adherence [54]. The MEDiterranean Islands Study (MEDIS) demonstrated that a high adherence to a Mediterranean-style dietary pattern decreased the risk of obesity and obesity-related complications in 1190 old men and women from Greece and Cyprus [40]. Similar results were observed in a smaller sample of older adults from Cyprus [55]. The Prevention with Medi-Diet (PREDIMED)—Canarias Intervention Randomized Trial tested the effects of three dietary regimens (i.e., Medi-Diet plus extra virgin olive oil, Medi-diet plus nuts, and a low-fat diet without a fixed daily caloric intake) on body composition in 351 community-dwelling individuals aged 55 to 80 years with type 2 diabetes or at least three cardiovascular risk factors [56]. After one year of intervention, participants allocated to Medi-Diet regimens experienced significant reductions in body adiposity and excess weight that were not observed in those on a low-fat diet [56]. In all these studies, obesity and high adiposity were defined according to BMI values and direct measurements of body fat by bioimpedance analysis (BIA) or DXA, respectively. As mentioned earlier, BMI may not be sufficiently sensitive for identifying obesity [8,9], with high misclassification rates observed for commonly adopted BMI cutoff points. On the other hand, BIA and especially DXA may not be available in settings with limited resources.

The positive effects of Medi-Diet on body fat accumulation may arise from its high content in plant-based food and a low consumption of meat and dairy products [39]. While ultra-processed foods elicit a high glycemic load and lower satiety, legumes, fruit, vegetables, and whole grains provide a higher intake of dietary fiber and low-density energy, and induce greater satiety [57]. Satiety-inducing properties of fiber depend on different mechanisms, such as increased mastication time and gastric distension, and stimulation of the release of appetite-reducing hormones, including cholecystokinin, glucagon-like peptide 1, and peptide YY [39]. Olive oil and fish provide mono- and polyunsaturated fatty acids with anti-inflammatory and antioxidant properties [58]. Moreover, when compared with saturated fats, unsaturated fatty acids increase energy expenditure, fat oxidation, and diet-induced thermogenesis [59,60]. A regular use of olive oil is not associated with weight gain, despite being one of the most energy-dense foods [61,62]. Unexpectedly, participants with low Medi-Diet adherence had lower daily energy intake. This finding may be due to a selective underreporting of “unhealthy foods”, such as those rich in carbohydrates and fats [63]. Energy underreporting is also more frequent in individuals living with obesity [64]. Vegetables, fruit, and fish instead tend to be reported to a similar extent by under-reporters and non-under-reporters [65].

Medi-Diet may also increase the diversity of gut microbiota and shape its composition toward an increase in probiotic species (e.g., Lactobacilli, Bifidobacteria) [66]. Such changes in the intestinal microbial ecosystem are associated with reduced inflammatory and oxidative status and amelioration in overall metabolic health [67]. The adoption of a Medi-Diet may also help manage obesity and high adiposity in older adults. Aged individuals are at increased risk of protein–energy malnutrition, which leads to weight loss and predisposes to negative health outcomes, including the development and/or worsening of sarcopenia and frailty [68]. Overweight and obesity have traditionally been considered protective in old age, as meta-analyses showed a lower mortality risk in healthy older adults in the overweight range [7,69,70]. A recent systematic review found a lower risk of short- (within 12 months) or long-term mortality (five years or more) in older adults living with overweight or obesity in 30 out of 58 studies [11]. The survival advantage was more evident in those with chronic diseases or experiencing an acute medical event as well in the oldest old [11]. In this regard, studies have shown that the risk of death and complications associated with excess body weight is greater in those aged 65–75 years than in younger adults but is lower in older individuals [71,72]. This phenomenon may be due to a “bottleneck” effect that leads a greater percentage of obese individuals to die early, while those who live longer may be the least compromised [73]. Alternatively, the “obesity paradox” might reflect greater robustness or higher reserves, and reduced risk of undernutrition [11]. However, the obesity paradox has been challenged by studies suggesting possible methodological errors and inaccurate assessments [74,75]. A meta-analysis by the Global BMI Mortality Collaboration conducted on data from 239 studies in four continents highlighted a consistent association of excess body weight with all-cause mortality [7]. Furthermore, as pointed out in a meta-analysis by Yuan et al. [76], obesity is associated with frailty as much as undernutrition, and high adiposity in older adults has been associated with higher inflammation levels and oxidative stress [77], metabolic abnormalities [78,79], worsening of sarcopenia and osteoporosis [77,80], poor physical performance [80,81], frailty [82], disability [83], and reduced quality of life [81,84]. On the other hand, the Medi-Diet has shown benefits on body adiposity [55], bone health [31,85], muscle mass and strength [31,86], as well as physical performance [29]. Moreover, adiposity is a key parameter in cardiovascular risk assessment. A systematic review by Kastorini et al. [87] found that the beneficial effects conveyed by a Medi-Diet on weight gain and obesity play a key role in protecting against coronary artery disease.

Although the present study reports interesting results, it has limitations that should be mentioned. First, the cross-sectional design prevents from drawing cause-and-effect conclusions between adherence to a Medi-Diet and body adiposity. Second, a substantial number of participants were excluded from the analysis because of incomplete data. Although efforts were made to maintain the duration of assessments within a reasonable length, participants were evaluated while attending social events or carrying out shopping activities. In several instances, participants decided to interrupt the assessment before all data could be collected. However, the main characteristics of participants with incomplete data did not significantly differ from those included in the analysis. This, together with the large sample size, limited the impact of participant exclusion on the robustness of results. Third, although current evidence indicates that the Woolcott and Bergman’s equation provides reliable RFM estimates across ages and ethnicities, it is not a direct measure of body fat. Nevertheless, RFM was shown to be a reliable predictor of whole-body fat percentage measured by DXA [15]. Furthermore, RFM has recently been included in the Moli-sani risk score [88], a validated algorithm to estimate the impact of modifiable risk factors on cardiovascular risk. Fourth, no data on alcohol consumption were collected. Fifth, data available in the Lookup 7+ database did not include information on disease conditions, medications, living arrangement, or socioeconomic status. Collection of a comprehensive personal and medical/drug history would significantly lengthen the assessments, rendering them unsuitable for the nontraditional contexts in which the research is conducted. This limitation, which is intrinsic to the Lookup 7+ survey, needs to be addressed in future studies specifically designed to evaluate the influence of personal and clinical variables on the association between dietary regimens and body adiposity. Sixth, virtually all participants were Caucasians; thereby, results may not be applicable to other ethnicities. Seventh, our study exclusively focused on older adults residing in the community. As a result, the implications and conclusions drawn from our findings may not be applicable to different contexts or populations. Eighth, there are several validated questionnaires available for evaluating adherence to the Medi-Diet. However, no single measure demonstrated clear superiority over others. Finally, the health benefits associated with the Medi-Diet are believed to be conveyed by a combination of nutritional factors as well as lifestyle and sociocultural influences, including engaging in regular physical activity, adequate rest, and conviviality [89]. Additional research is required to determine whether the Medi-Diet as a whole lifestyle pattern or its dietary components alone affect the risk of body fat accumulation.

## 5. Conclusions

The results obtained from this study indicate that there is a significant correlation between a higher Medi-Diet adherence and decreased levels of body adiposity in a large and relatively unselected sample of older adults residing in the community. Additional research is required to ascertain the possible impact of adopting a Mediterranean-style dietary pattern on general health and quality of life in older individuals.

## Figures and Tables

**Table 1:** Main characteristics of study participants according to their adherence to a Mediterranean diet.

	Total Sample (*n* = 2092)	Mediterranean Diet Adherence			*p*
		Low (*n* = 413)	Moderate (*n* = 1198)	High (*n* = 481)	
Age, years	73.1 (5.9)	73.1 (6.0)	73.2 (5.9)	73.1 (5.8)	0.929
Sex, female	1117 (53.4%)	240 (58.1%)	611 (51.0%) ^a^	266 (55.3%)	0.028
Active smoking	315 (15.1%)	71 (17.2%)	189 (15.8%)	55 (11.4%) ^a,b^	0.034
Physically active	1118 (53.4%)	203 (49.2%)	652 (54.4%)	263 (54.7%)	0.149
Energy intake, kcal/day	1410 (264)	1160 (244)	1410 (240) ^a^	1586 (179) ^a,b^	<0.001
Medi-Lite score	10.4 (1.9)	7.5 (0.8)	10.1 (0.8) ^a^	12.8 (1.0) ^a,b^	<0.001
BMI, kg/m^2^	25.9 (3.93)	26.5 (4.11)	26.0 (3.79)	25.4 (4.03) ^a,b^	<0.001
Waist circumference, cm					
Women	88.8 (12.1)	90.5 (11.8)	89.5 (12.4)	86.4 (11.6) ^a,b^	<0.001
Men	98.9 (10.0)	101 (10.1)	98.9 (9.9) ^a^	97.5 (10.1) ^a^	0.002
RFM, %					
Women	39.6 (5.14)	40.6 (4.86)	40.0 (5.14)	38.5 (5.03) ^a,b^	<0.001
Men	29.0 (3.58)	29.9 (3.35)	29.0 (3.57)	28.4 (3.66) ^a,b^	<0.001
High adiposity	971 (46.4%)	224 (54.2%)	556 (46.4%) ^a^	191 (39.7%) ^a,b^	<0.001

Data are reported as absolute numbers (percentages) for categorical variables (sex, active smoking, physically active, high adiposity). Continuous variables are shown as means (standard deviations). Physically active: engaged in physical activity at least twice weekly, with each session lasting a minimum of 30 min, throughout the preceding year. High adiposity: relative fat mass ≥40% in women and ≥30% in men. ^a^ *p* < 0.05 vs. low adherence; ^b^ *p* < 0.05 vs. moderate adherence. Abbreviations: BMI, body mass index; RFM: relative fat mass.

**Table 2:** Unadjusted and adjusted association between adherence to a Mediterranean diet and high body adiposity.

Characteristics	Unadjusted OR (95% CI)	*p*	Model 1 OR (95% CI)	*p*	Model 2 OR (95% CI)	*p*
Adherence to Mediterranean diet						
High adherence	Reference (1.00)	-	Reference (1.00)	-	Reference (1.00)	-
Moderate adherence	1.31 (1.06–1.63)	0.013	1.34 (1.08–1.67)	0.008	1.36 (1.09–1.71)	0.007
Low adherence	1.80 (1.38–2.35)	<0.001	1.80 (1.38–2.35)	<0.001	1.85 (1.33–2.58)	<0.001
Age			1.03 (1.02–1.05)	<0.001	1.03 (1.01–1.04)	0.001
Sex, female			1.50 (1.26–1.78)	<0.001	1.47 (1.23–1.76)	<0.001
Active smoking					1.01 (0.78–1.29)	0.990
Daily energy intake					1.00 (0.99–1.01)	0.400
Physically active					0.48 (0.40–0.58)	<0.001

High body adiposity: relative fat mass ≥40% in women and ≥30% in men. Model 1: odds ratio adjusted for age and female sex; model 2: odds ratio adjusted for age, female sex, active smoking, and engagement in physical activity. Abbreviations: CI: confidence interval; OR: odds ratio.

## Data Availability

The data that support the findings of this study are available on request from the corresponding author. The data are not publicly available due to their containing information that could compromise the privacy of the research participants.

## Supplementary Materials

The following supporting information can be downloaded at: https://www.mdpi.com/article/10.3390/nu15234892/s1, Lookup 7+ Project Team Members.
