# Supplementary material for: Lower Adherence to a Mediterranean Diet Is Associated with High Adiposity in Community-Dwelling Older Adults: Results from the Longevity Check-Up (Lookup) 7+ Project
Source: Nutrients. 2023 Nov 23;15(23):4892. doi: 10.3390/nu15234892 (PMC10708281; doi:10.3390/nu15234892)
Supplement: Supplementary file 1 [file nutrients-15-04892-s001.zip › nutrients-2707044-supplementary.pdf]

Lookup 7+ Project Team Members

Clara Agostino, Fiorella Ambrosio, Andrea Bellieni, Roberto Bernabei, Andrea Berti, Damiano Biscotti, Vincenzo Brandi, Maria Modestina Bulla, Stefano Cacciatore, Riccardo Calvani, Camilla Cocchi, Lucio Catalano, Francesca Ciciarello, Giuseppe Colloca, Luca Colavita, Francesco Paolo Damiano, Mariaelena D'Elia, Federica D'Ignazio, Domenico Fusco, Daniele Elmi, Sofia Fabrizi, Francesco Pio Fontanella, Raffaele Forino, Giuseppe Gallo, Vincenzo Galluzzo, Giordana Gava, Tommaso Giani, Giulia Giordano, Rossella Giordano, Francesca Giovanale, Silvia Ialungo, Rosangela Labriola, Francesco Landi, Chiara Leone, Elena Levati, Myriam Macaluso, Laura Macculi, Luca Mariotti, Luca Marrella, Anna Maria Martone, Emanuele Marzetti, Claudia Massaro, Rossella Montenero, Maria Vittoria Notari, Cristina Pais, Martina Persia, Anna Picca, Flavia Pirone, Simona Pompei, Rosa Ragozzino, Carla Recupero, Antonella Risoli, Sara Rocchi, Alessandra Rocconi, Elisabetta Rota, Giulia Rubini, Andrea Russo, Sara Salini, Giulia Savera, Elisabetta Serafini, Sofia Simoni, Chiara Taccone, Elena Tagliacozzi, Roberta Terranova, Matteo Tosato, Salvatore Tupputi, Maria Beatrice Zazzara, Maria Zuppardo.
